# G protein‐coupled receptor kinase‐2: A potential biomarker for early diabetic cardiomyopathy

**DOI:** 10.1111/1753-0407.12991

**Published:** 2019-11-03

**Authors:** Shuiqing Lai, Xiaoying Fu, Shufen Yang, Shuting Zhang, Qiuxiong Lin, Mengzhen Zhang, Hongmei Chen

**Affiliations:** ^1^ Department of Endocrinology, Guangdong Provincial People's Hospital / Guangdong Academy of Medical Sciences Guangdong Provincial Geriatrics Institute Guangzhou P. R. China; ^2^ Shantou University Medical College Shantou P. R. China; ^3^ Guangdong Provincial Key Laboratory of Clinical Pharmacology Guangdong Cardiovascular Institute Guangzhou P. R. China

**Keywords:** diabetic cardiomyopathy, GRK2, left ventricular diastolic dysfunction, myocardium, peripheral blood mononuclear cells, 糖尿病心肌病, G蛋白偶联受体激酶2, 左心室舒张功能不全, 心肌组织, 外周血单核细胞

## Abstract

**Background:**

G protein‐coupled receptor kinase‐2 (GRK2) has been shown as a key regulator of cardiac function, and the myocardial GRK2 levels are mirrored by the levels in peripheral blood mononuclear cells (PBMCs). In this study, we evaluated the myocardial and PBMCs GRK2 levels in early diabetic cardiomyopathy (DCM).

**Methods:**

C57BL/KS‐db/db male diabetic mice at 12 weeks of age, as the type 2 diabetes (T2DM) animal model of early DCM were evaluated. Forty‐four T2DM patients with left ventricular diastolic dysfunction (LVDD), without evidence of hypertension, coronary artery diseases, congestive heart failure, and diabetic complications and without evidence of ischemia in a maximal treadmill exercise test, were recruited as the DM + LVDD group; 30 age‐matched T2DM patients without LVDD were recruited as the DM control group. Left ventricular diastolic function was evaluated by cardiac tissue Doppler. The pseudonormal pattern of ventricular filling and E′/A′ < 1 were regarded as LVDD.

**Results:**

Compared to 8‐week‐old diabetic mice and 12‐week‐old control mice, GRK2‐mRNA level and expression in myocardial tissues of 12‐week‐old diabetic mice were significantly increased, as well as the left ventricular wall thickness and systolic function. And the collagen volume fraction (CVF), collagen‐3 expression, P53 expression, and cell apoptotic rate in the myocardium of 12‐week‐old diabetic mice elevated as well. The GRK2‐mRNA level in PBMCs of DM with LVDD was significantly higher than in DM control without LVDD.

**Conclusions:**

GRK2 expression increased in the myocardial tissue and the PBMCs at the early stage of DCM. These data support further research on the role of GRK2 as the clinical biomarker for early DCM.

## Graphical abstract

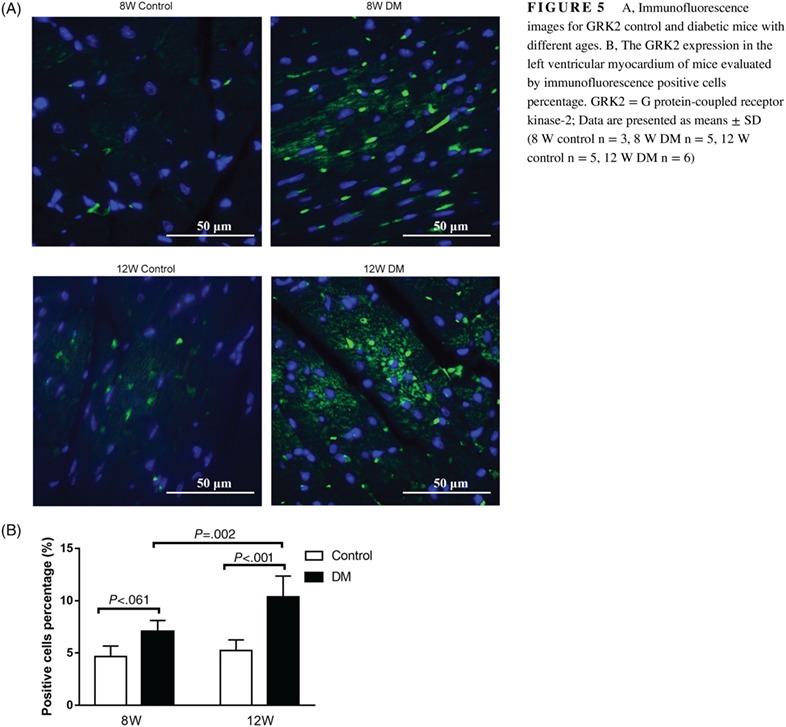

**Highlights**

- The GRK2 level elevated not only in the myocardium of diabetic mice with early stage of diabetic cardiomyopathy but also in the peripheral blood mononuclear cells of type 2 diabetic patients with the early stage of diabetic cardiomyopathy.
- The study findings suggested that the GRK2 was a potential biomarker for early diabetic cardiomyopathy.

## 摘要

**背景:**

研究显示G蛋白偶联受体激酶2(GRK2)是调节心脏功能的关键因子, 而心肌GRK2的表达水平与外周血单核细胞(PBMC)的GRK2表达存在一致性。本研究评估了早期糖尿病性心肌病(DCM)PBMC的GRK2水平。

**方法:**

动物研究以12周龄的C57BL/KS‐db/db雄性糖尿病小鼠作为早期DCM的2型糖尿病(T2DM)研究模型。人群研究中招募了44位T2DM伴有左心室舒张功能障碍(LVDD)的患者为DM + LVDD组, 他们均排除了高血压、冠状动脉疾病、充血性心力衰竭和糖尿病并发症等疾病, 并且在心脏运动负荷测试中没有缺血迹象。另外招募 30位年龄相匹配的无LVDD的T2DM患者为DM对照组。左心室舒张功能通过心脏组织多普勒评估。心室假性正常化充盈和E’/A’ <1定义为LVDD。

**结果:**

本研究结果显示, 与8周龄糖尿病小鼠和12周龄对照小鼠相比, 12周龄糖尿病小鼠心肌组织中GRK2‐mRNA水平及其表达明显增加, 且左心室壁厚度增加和收缩功能增强。胶原蛋白的体积分数(CVF)、胶原蛋白3、P53表达水平和细胞凋亡率在12周龄糖尿病小鼠心肌中也明显升高。人群研究显示, 与DM对照组患者相比, DM+LVDD组患者PBMC中GRK2‐mRNA水平明显升高。

**结论:**

在DCM的早期阶段, 心肌组织和PBMC的GRK2水平增加。这些数据支持进一步探讨GRK2在早期DCM诊断中作为临床标志物的可能性。

## 1 INTRODUCTION

Type 2 diabetes (T2DM) is a serious worldwide health problem with a striking increase in prevalence and incidence. Heart failure (HF) due to cardiovascular disease is the major etiological factor for death in diabetic patients; however, morbidity and mortality of HF are shown to increase significantly among diabetic patients without cardiovascular disease.^1^, ^2^ The Heart and Soul Study demonstrated that diabetes and glycemic control were independent risk factors for new‐onset HF in patients with stable coronary artery diseases who were free from HF at baseline.^3^ Additionally, the incidence of HF in patients with diabetes was 2~3‐fold higher than in those without diabetes.^4^

Cardiomyopathy induced by glucose metabolism disorder, which is defined as diabetic cardiomyopathy (DCM), may be a crucial reason for heart dysfunction. Cardiomyocyte apoptosis and myocardial fibrosis are the early major pathological changes of DCM. Although the exact prevalence of DCM is unknown, the incidence of diastolic dysfunction in patients with T2DM is up to 30% in some studies.^5^ Accumulating evidence suggests that left ventricular diastolic dysfunction (LVDD) is the earliest clinical manifestation of DCM, and LVDD implies cardiac tissue remodeling. Poirier and his colleagues found that about 60% of T2DM patients without hypertension and cardiovascular diseases were found with LVDD by cardiac tissue Doppler. E wave and A wave are defined as early and late ventricular filling velocities evaluated by cardiac tissue Doppler. Based on these studies, E'/A' value <1 and pseudo‐normal pattern of ventricular filling were regarded as LVDD.^6^

G‐protein‐coupled receptor kinase‐2 (GRK2), which localizes on the plasma membrane, has been shown as a key regulator of cardiac contractile function. Early data have suggested that the myocardial GRK2 levels were mirrored by levels in peripheral blood mononuclear cells (PBMCs).^7^, ^8^, ^9^ Additionally, GRK2 was upregulated in pathological situations such as HF, hypertrophy, and hypertension. What is more, lymphocyte GRK2 protein levels can independently predict prognosis in patients with HF.^10^, ^11^ Another study showed that lymphocyte GRK2 levels increased during acute myocardial infarction and were associated with worse cardiac function. This study indicated that GRK2 could be a biomarker and predictor of ventricular remodeling after myocardial infarction and could facilitate the tailoring of appropriate therapy for high‐risk patients.^12^ Moreover, the increase of lymphocytes GRK2 was observed in HF patients with DM compared to non‐DM.^13^ Our previous observational study demonstrated that GRK2 expression increased in lymphocytes of T2DM patients without evidence of hypertension and coronary artery diseases.^14^ Additionally, we found that the GRK2‐mRNA level was concentration‐dependently increased in H9C2 cardiomyoblasts cultured with high glucose medium, indicating that hyperglycemia induced GRK2 upregulation.^15^ However, the GRK2 expression in the early stage of DCM remains unclear.

Db/db mouse is a model of T2DM with leptin receptor insensitivity characterized by significant obesity, fasting hyperglycemia, hyperinsulinemia, and insulin resistance. It also develops changes in cardiac structure and function similar to those in T2DM patients.^16^ Recent studies have confirmed that functional and morphological alterations in db/db hearts are consistent with DCM.^17^, ^18^

In this study, we aimed to investigate the association of GRK2 with early DCM by estimating the expression of GRKs both in the myocardium of diabetic mice and in the PBMCs of patients with early DCM.

## 2 MATERIALS AND METHODS

### 2.1 Animal study

#### 2.1.1 Animal preparations and experimental protocol

Eleven diabetic male mice of the [C57BL/ks (db/db)] strain and eight nondiabetic congenic littermates at 7 week of age were purchased from Nanjing Biomedical Research Institute of Nanjing University. Both diabetic mice and control mice were randomized into two groups after purchase (5 for 8 W DM group, 6 for 12 W DM group, 3 for 8 W control group, and 5 for 12 W control group). All mice were housed under constant conditions of temperature (22°C) and humidity (55%), with a 12:12‐hours reversed light:dark cycle and had free access to food and water. Food and water intake was recorded daily and food and water were replenished every 2‐3 days according to the intake condition. Mice were weighed and the blood glucose concentration was measured from the tail vein every week using a OneTouch Blood Glucose Monitoring glucometer (glucose assay kit; Johnson & Johnson, New Brunswick, NJ). Cardiac function of mice was evaluated by echocardiography at 8 or 12 weeks of age, after they were euthanized; meanwhile, body weight was determined before the anatomy; hearts were harvested, rinsed briefly in phosphate‐buffered saline, blotted dry, and weighed. The left ventricles were sectioned for hematoxylin‐eosin (HE) staining, Masson staining, TUNEL staining, western blot, and real‐time quantitative polymerase chain reaction (PCR) assays. Animal care and experiments conformed to the Guide for the Care and Use of Laboratory Animals, approved by the US National Institutes of Health (eighth Edition, National Research Council, 2011), and were also approved by the Research Ethics Committee of Guangdong Provincial People's Hospital (No. GDREC2014170A).

#### 2.1.2 Echocardiographic evaluation

The mice underwent transthoracic two‐dimensional guided M‐mode echocardiography using a Technos MPX ultrasound system (ESAOTE, Genoa, Italy). M‐mode images of the left ventricle were obtained from parasternal short‐axis and the parasternal long‐axis view. The M‐mode images measured systolic and diastolic wall thickness and left ventricles end‐systolic diameters (LVESD) and end‐diastolic diameters (LVEDD) respectively. Left ventricles fractional shortening (FS) was calculated as FS = (LVEDD‐LVESD)/LVESD; Left ventricular ejection fraction (EF) was calculated as EF = (End‐diastolic volume − End‐systolic volume)/ End‐diastolic volume. End‐diastolic volume and End‐systolic volume were calculated by the Teichholz formula: volume = 7/(2.4 + left ventricular diameter) + left ventricular diameter.^3^

#### 2.1.3 HE and Masson staining

After fixation with 10% formaldehyde, the cardiac tissues were subjected to dehydration and embedded in paraffin. Tissue section 4 um in thickness were deparaffinized, rehydrated, and stained with hematoxylin and eosin to evaluate the cardiac structure. Other 4 um‐thick serial sections were subjected to Masson staining to observe the degree of myocardial fibrosis. The interstitial collagen volume fraction (CVF) was calculated as the area occupied by the blue‐ dyed tissue, divided by the total myocardial area under direct vision. CVF was calculated by Image Pro Plus software.

#### 2.1.4 TUNEL staining

Heart tissues was fixed in 10% formaldehyde, embedded in paraffin, and then sectioned at 4 um. The Meilun One Step TUNEL Apoptosis assay Kit (Dalian Meilun Biotechnology, Dalian, China, Catalog number MA0223) was used according to the manufacturer's instructions and details about the TUNEL staining assay have been published in our previous article.^14^ The apoptotic cells were characterized by positive fluorescence detection. One thousand cells were counted, the positive cells were identified, counted, and analyzed. The apoptotic index was expressed as the percentage of positive cells (Nikon 80i, Tokyo, Japan).

#### 2.1.5 Immunofluorescence assay

Heart tissue were fixed in 10% formaldehyde, embedded in paraffin, and then sectioned at 4 um. Heat the unstained slides in an oven at 60°C for 45 minutes, rehydrated the slides through two changes of xylene and a graded alcohol series, and then rinsed the slides in dH2O and PBS. The slides were permeabilized with 0.1% Triton X‐100 and blocked with the goat serum/PBS (1:200) for 30 minutes at room temperature. After that, the slides were incubated with GRK2 antibody (Santa Cruz Biotechnology, Dallas, TX, Catalog number: sc‐13143) for 1 hour at room temperature. Following that, the slides were washed with PBS once and then incubated with a goat antirabbit IgG 594 for 30 minutes at room temperature. Nuclei were stained with DAPI for 5 minutes. The images were captured using a Nikon 80i microscope. Positive cell percentage was expressed as the number of immunofluorescence positive cells of all cardiomyocyte nuclei per field.

#### 2.1.6 Western blot analysis

After extraction of myocardial proteins, equal amounts of the protein preparations were separated by 10% sodium dodecyl sulfate‐polyacrylamide gel electrophoresis. The separated proteins were transferred to polyvinylidene fluoride membranes for 120 minutes at 200A. The membrane was blocked with 5% milk in Tris‐buffer saline solution containing 0.05% Tween‐20 and then incubated with a primary antibody against collagen‐1 (1:1000, Protein tech), collagen‐3 (1:1000, Protein tech), P53(1:2000, Protein tech), and glyceraldehyde 3‐phosphate dehydrogenase (GAPDH; 1:5000, Protein tech) at 4°C overnight. The following day, the membrane was incubated with horseradish peroxidase‐conjugated secondary antibody for 2 hours at 4°C. The immunoreactive proteins were visualized using ECL plus detection system (GE Healthcare, Waukesha, WI).

#### 2.1.7 Real‐time quantitative PCR

Total RNAs were extracted from myocardial tissues using Trizol reagent (Ambion, Austin, TX). First‐strand cDNAs were synthesized using 5X Prime Script RT Master Mix (Takara Biomedical Technology Co., China), according to the manufacturer's protocol. The real‐time PCR was performed using CFX Connect^TM^ Real‐Time System (Bio‐Rad, Hercules, CA). One microliter of RT reaction products were amplified by PCR in a volume of 10 ul under the following conditions, 95°C for 2 minutes, followed by 40 cycles of 95°C for 5 seconds, 60°C for 30 seconds, using iTaq Universal SYBR Green Supermix (Bio‐Rad). Primers specific for GAPDH (sense primer: 5′‐AAG AAG GTG GTG AAG CAG GC‐3′, antisense primer:5′‐TCC ACC ACC CTG TTG CTG TA‐3′), GRK2 (sense primer: 5′‐GAC ACT TGC GTT CCT TGA T − 3′, antisense primer: 5′‐GCG GCG ATA CTT CTA CTT G‐3′), GRK3 (sense primer: 5′‐GCA CTT CAT GGC ATA CAT T − 3′, antisense primer: 5′‐GAA ACA GGT GAC GGC TAC‐3′), and GRR5 (sense primer: 5′‐AGG CCA GTC ACC ATT TCG AG −3′, antisense primer: 5′‐GCA GAT GGA CTT GGC CTC TT‐3′) were synthesized. To normalize RNA content, the GAPDH served as the internal control. Each sample was amplified in triplicate and normalized vs the endogenous control. Results were calculated using the 2^−ΔΔCt^ method.

### 2.2 Patient study

#### 2.2.1 Patients and echocardiographic evaluation

T2DM patients aged 30‐60 years with LVDD were recruited in this study, including 22 men and 22 women. Thirty T2DM patients without LVDD were recruited as the DM control group in the study. All of them were without evidence of hypertension, coronary artery diseases, congestive heart failure, and diabetic complications and without evidence of ischemia in a maximal treadmill exercise test; and no other medications than antidiabetics were used by all patients. All patients included used oral antihyperglycemic medications, single medication, or combination of the following medications: metformin, acarbose, and rosiglitazone. LVDD was evaluated by cardiac tissue Doppler and contractile function was evaluated by echocardiography in all subjects. The pseudonormal pattern of ventricular filling and E′/A′ < 1 regarded as DM + LVDD, and 0.8 < E′/A′ < 1 was DM + LVDD1 group, the pseudonormal pattern of ventricular filling and E′/A′ < 0.8 was DM + LVDD2 group. The clinical investigation was conducted according to the principles expressed in Declaration of Helsinki, and approved by the Research Ethics Committee of Guangdong Provincial People's Hospital (Guangzhou, China), approval number GDREC [2009] 101. All subjects gave written consent to participate in this study and were told to follow only conventional medical treatments without additional burden.

#### 2.2.2 Real‐time quantitative PCR

Blood samples were taken from all patients after 10 hours fasting by using Ficoll‐Paque density gradient centrifugation (GE Healthcare Life Sciences, Pittsburgh, PA) and then centrifuged for 20 minutes at 2000 rpm, and the peripheral blood mononuclear cell layer was collected according to the instruction.^19^ Total RNA was isolated from PBMCs using Trizol Reagent. The quality and quantity of the isolated RNA were determined before reverse transcription. Reverse transcription was performed by using the Thermo Script RT‐PCR system for experimental and control sample respectively. Primer specific for GAPDH (sense primer: 5′‐TCC ATG ACA ACT TTG GTA TCG T‐3′, antisense primer:5′‐GTG GGC CAT GAG GTC CAC‐3′); hGRK2 (sense primer: 5′‐CAT TCA TGG TCA GGT GGA TG‐3′, antisense primer:5′‐TTC TCG AAG AGT GCC ACT G‐3′). The first‐strand DNA was used to amplify the following genes fragment: GRK2 and GAPDH by PCR. The specificity and size of the PCR products were tested by running it on a 1.5% agarose gel.

#### 2.2.3 Clinical measurements

Glucose level was determined by the glucose oxidation method (Synchron systems LX20). Cholesterol and triglycerides were determined by enzymatic methods (Synchron systems LX20). HbA1c was measured by Bio‐Rad DiaSTAT system (Bio‐Rad Laboratories Inc. CA. USA，Catalog number 210‐0002).

### 2.3 Statistical analysis

All data were presented as means ± SD. Statistical analyses were performed with SPSS 20.0 software, for animal study two‐way analysis of variance (genotype x age) were performed and followed by Bonferroni post hoc test. For patients study one‐way analysis of variance was performed and followed by LSD post hoc analysis for multiple comparisons; the GRK2 difference of human PBMCs between groups were tested by using analysis of covariance, age, and HbA1C were adjusted and followed by LSD post hoc analysis. *P* value <.05 was considered statistically significant.

## 3 RESULTS

### 3.1 General characteristics and echocardiographic analysis of mice

**Figure 1:**
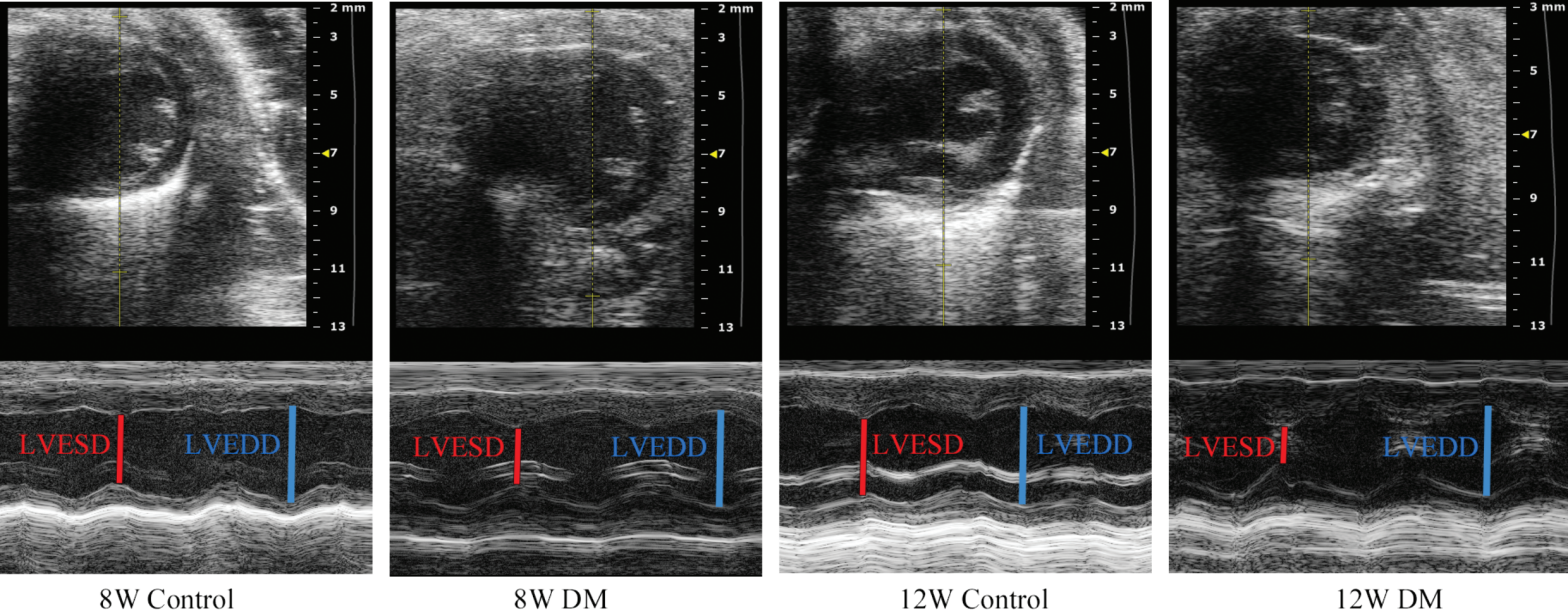
Echocardiographic images demonstrating an example of left ventricular diameter in M‐mode. LVESD, Left ventricular end‐systolic diameters; LVEDD, Left ventricular end‐diastolic diameters

By echocardiography, mean heart rate was lower in 12‐week‐old diabetic mice than 12‐week‐old control mice whereas no significant differences in mean heart rate between diabetic mice and control mice at 8 week of age. Left ventricular anterior/posterior wall at systole and left ventricular anterior wall at diastole were significantly thickening in 12‐week‐old diabetic mice, compared to 8‐week‐old diabetic mice and 12‐week‐old control mice. EF and FS were also increased significantly in 12‐week‐old diabetic mice compared to 8‐week‐old diabetic mice and 12‐week‐old control mice. And there was no significant difference in EF and FS between the 8‐week‐old diabetic mice and 8‐week‐old control mice. (Table 1). Calculation of left ventricular EF and FS by M mode was showed in Figure 1. The parasternal long‐axis view revealed that left ventricular end‐systolic diameter was significantly reduced in 12‐week‐old diabetic mice compared to other groups. (Table 1 and Figure 1).

**Table 1:** Echocardiographic characteristics of control mice and diabetic db/db mice

	8 W Control	8 W db/db	12 W Control	12 W db/db
N	3	5	5	6
Body weight (g)	20.74 ± 0.79	39.61 ± 2.37*	22.45 ± 0.92**	44.46 ± 3.11* ^,^ ** ^,^ ***
Blood glucose (mmol/L)	11.30 ± 0.99	26.22 ± 3.93*	11.04 ± 0.50**	29.00 ± 3.94* ^,^ ***
Heart rate (bpm)	389 ± 58	407 ± 46	572 ± 26* ^,**^	509 ± 60* ^,^ ** ^,^ ***
Heart weight (mg)	97.45 ± 1.23	128.08 ± 20.00	127.46 ± 23.09	128.72 ± 7.02
LVAWs (mm)	1.01 ± 0.18	1.23 ± 0.23	1.29 ± 0.05	1.70 ± 0.12* ^,^ ** ^,^ ***
LVAWd (mm)	0.75 ± 0.00	0.79 ± 0.21	0.92 ± 0.15	1.13 ± 0.13** ^,^ ***
LVPWs (mm)	0.96 ± 0.05	1.19 ± 0.21*	1.16 ± 0.07	1.41 ± 0.08* ^,^ ** ^,^ ***
LVPWd (mm)	0.73 ± 0.03	0.89 ± 0.20	0.84 ± 0.09	0.97 ± 0.13
LVESD	2.23 ± 0.69	2.24 ± 0.41	2.36 ± 0.53	1.53 ± 0.22** ^,^ ***
LVEDD	3.42 ± 0.49	3.29 ± 0.58	3.41 ± 0.52	2.93 ± 0.20
FS (%)	35.6 ± 11.0	32.0 ± 4.9	31.1 ± 5.4	47.8 ± 4.1** ^,^ ***
EF (%)	65.2 ± 15.3	61.2 ± 6.9	59.7 ± 8.4	80.4 ± 4.5** ^,^ ***

*Note:* Data are presented as means ± SD.

Abbreviations: EF, Ejection fraction; FS, Fractional shortening; LVAWd, Left ventricular anterior wall thickness at diastole; LVAWs, Left ventricular anterior wall thickness at systole; LVEDD, Left ventricular end‐diastolic diameters; LVESD, Left ventricular end‐systolic diameters; LVPWd, Left ventricular posterior wall thickness at diastole; LVPWs, Left ventricular posterior wall thickness at systole.

* *P* < .05, vs 8 W Control group

** *P* < .05, vs 8 W db/db group

*** *P* < .05, vs 12 W Control group.

### 3.2 Diabetes‐induced fibrosis

**Figure 2:**
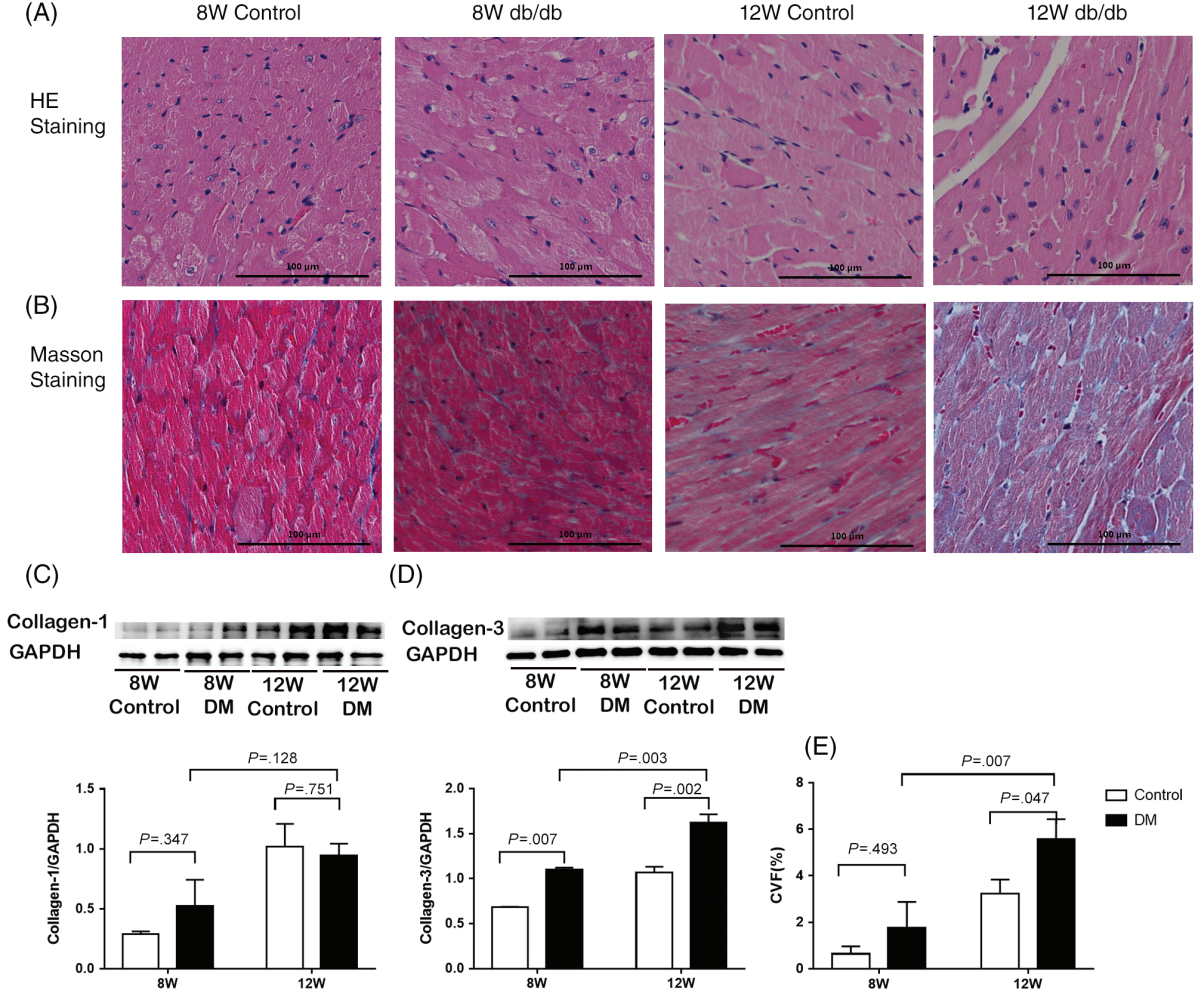
A, HE staining of the left ventricular myocardium from 8 W/12 W control group and 8 W/12 W diabetic group. B, Masson staining of the left ventricular myocardium from 8 W/12 W control group and 8 W/12 W diabetic group. C, Left ventricular myocardium collagen‐1 protein expression. D, Left ventricular myocardium collagen‐3 protein expression. E. Left ventricular myocardium CVF. Scale bar =100 μm applies to all images. CVF, Collagen volume fraction. Data are presented as means ± SD (8 W control n = 3, 8 W DM n = 5, 12 W control n = 5, 12 W DM n = 6)

Cardiac muscle fibers were disordered, cell gap increased, and many of the fibers were collapsed in the myocardium of 12‐week‐old diabetic mice, according to the HE staining (Figure 2A). Masson staining detected an elevated deposition of extracellular matrix within the interstitial and mainly perivascular areas of the myocardium of 12‐week‐old diabetic mice (Figure 2B). The CVF was significantly increased in 12‐week‐old diabetic mice compared to 8‐week‐old diabetic and 12‐week‐old control mice. Protein expression profiles showed a significant increase in collagen‐3 in 8‐week‐old and 12‐week‐old diabetic mice compared to the corresponding age control group, however no significant changes in collagen‐1 expression between different groups (Figure 2C,D).

### 3.3 Cardiomyocyte apoptosis

**Figure 3:**
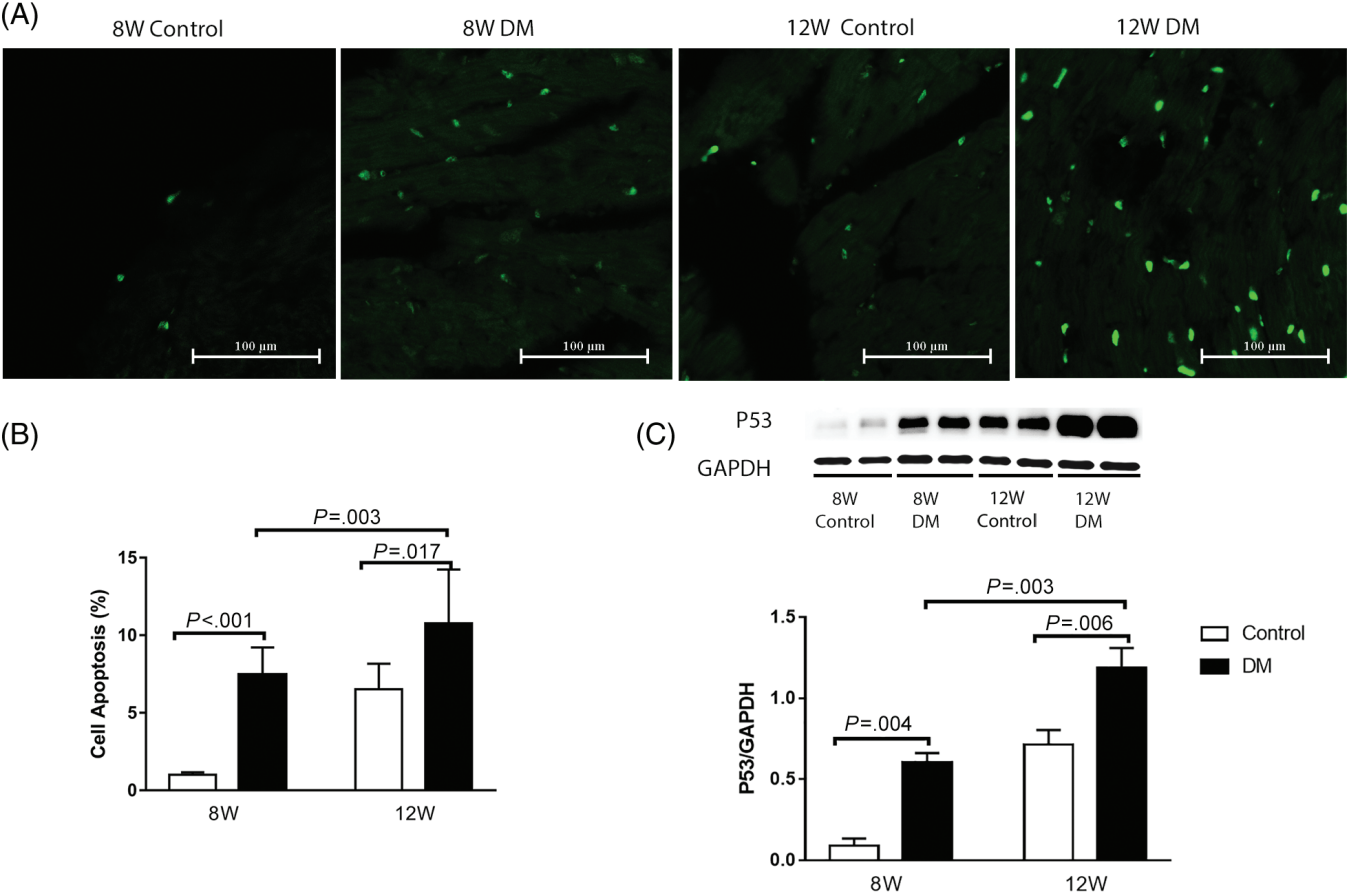
A, TUNEL staining under an inverted fluorescence microscope B, Cell apoptosis rate evaluated by TUNEL. C, The expression of P53 in the left ventricular myocardium of mice. Data are presented as means ± SD (8 W control n = 3, 8 W DM n = 5, 12 W control n = 5, 12 W DM n = 6)

The cardiomyocyte apoptosis rate of diabetic mice, measured via TUNEL was significantly increased, compared to that of the corresponding age control group (Figure 3A,B). The western blotting showed that P53 expression was significantly increased in 12‐week‐old diabetic mice compared to the 8‐week‐old diabetic mice (Figure 3C).

### 3.4 The GRKs RNA level in the left ventricular myocardium of mice

**Figure 4:**
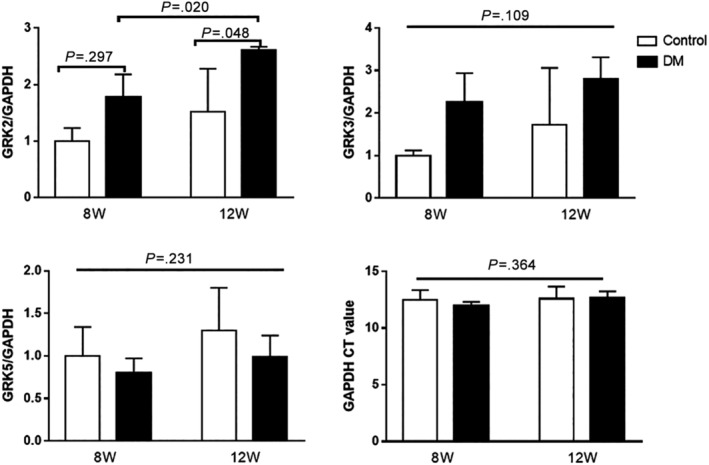
The mRNA level of GRK2, GRK3 and GRK5 in the left ventricular myocardium of mice. GRK2 = G protein‐coupled receptor kinase‐2; GRK3 = G protein‐coupled receptor kinase‐3; GRK5 = G protein‐coupled receptor kinase‐5. Data are presented as means ± SD (8 W control n = 3, 8 W DM n = 5, 12 W control n = 5, 12 W DM n = 6)

**Figure 5:**
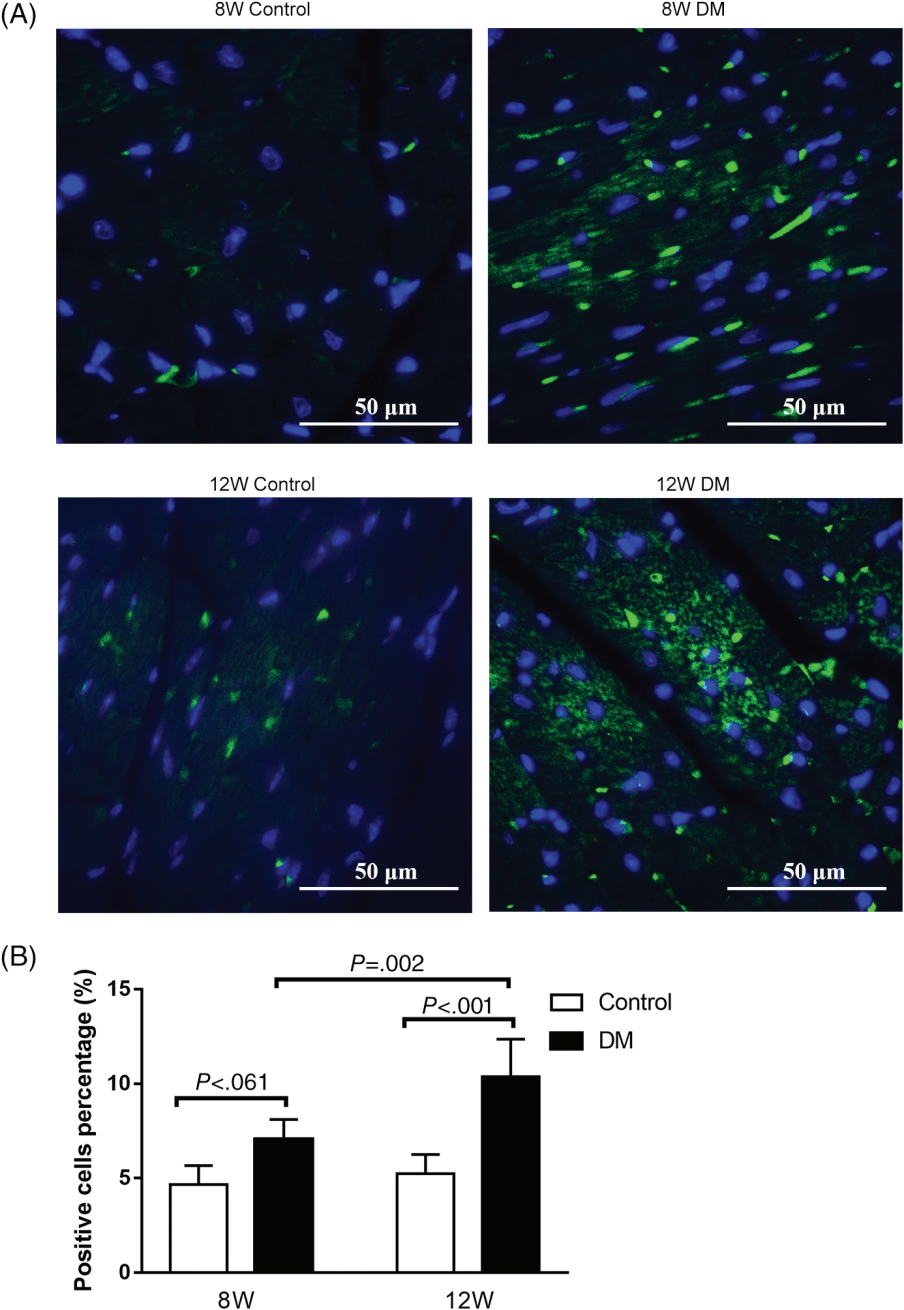
A, Immunofluorescence images for GRK2 control and diabetic mice with different ages. B, The GRK2 expression in the left ventricular myocardium of mice evaluated by immunofluorescence positive cells percentage. GRK2 = G protein‐coupled receptor kinase‐2; Data are presented as means ± SD (8 W control n = 3, 8 W DM n = 5, 12 W control n = 5, 12 W DM n = 6)

GRK2‐mRNA level was significantly elevated in 12‐week‐old diabetic mice compared to the 12‐week‐old control group and elevated in 8‐week‐old diabetic mice compared to the 8‐week‐old control group; however, the difference was not statistically significant (Figure 4); so as for the GRK2 expression in the left ventricular myocardium of mice evaluated by the immunofluorescence assay (Figure 5). There were no significant differences in GRK3‐mRNA level and GRK5‐mRNA level in the myocardium of mice between diabetic group and control group at both 8 and 12 week of age (Figure 4).

### 3.5 General characteristics and echocardiographic analysis of T2DM patients

There was no significant difference in plasma fasting glucose concentrations, body mass index, and HbA1c between the DM control and DM LVDD subjects (Table 2). Heart function was evaluated by echocardiography, the systolic function was normal in all patients (Table 2).

**Table 2:** General characteristics and echocardiographic analysis of T2DM patients

	DM control (*n* = 30)	DM + LVDD	
		DM + LVDD1 (n = 22)	DM + LVDD2(*n* = 22)
Age (Y)	44.43 (30‐57)	49.86 (32‐62)*	52.23 (37‐64)*
Gender (F/M)	15/15	11/11	11/11
Diabetes duration (Months)	33.2 (1‐144)	48.36 (1‐144)	44.23 (1‐240)
BMI (kg/m^2^)	23.33 ± 1.74	23.31 ± 2.45	23.90 ± 2.24
FBG (mmol/L)	9.54 ± 3.35	9.74 ± 2.85	9.07 ± 2.52
HbA1C (%)	9.40 ± 2.79	9.72 ± 2.59	9.15 ± 2.75
TG (mmol/L)	1.72 ± 1.21	1.39 ± 0.62	2.00 ± 1.49
LDL (mmol/L)	2.67 ± 0.87	2.67 ± 0.93	2.71 ± 1.02
LVDd (cm)	45.40 ± 3.44	43.77 ± 3.84	45.23 ± 3.01
LVDs (cm)	26.07 ± 2.74	26.59 ± 3.90	27.18 ± 2.65
LVPW	8.90 ± 0.84	9.27 ± 1.12	9.36 ± 1.29
EF (%)	66.60 ± 2.67	65.36 ± 2.90	66.86 ± 2.80

*Note:* Data are presented as means ± SD.

Abbreviations: BMI, Body mass index; EF, Ejection fraction.; FBG, Fast blood glucose; HbA1c, Hemoglobin A1c; LDL, Low density lipoprotein; LVDd, Left ventricular diameter at diastole; LVDD, Left ventricular diastolic dysfunction; LVDs, Left ventricular diameter at systole; LVPW, Left ventricular posterior wall thickness; TG, Triglyceride.

* *P* < .05, vs control group.

### 3.6 GRK2 expression in PBMCs of early DCM

**Figure 6:**
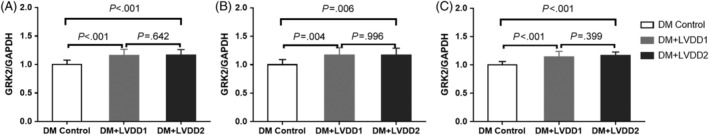
A, GRK2 mRNA expression in PBMCs of T2DM. B, GRK2 mRNA level in PBMCs of female T2DM. C, GRK2 mRNA level in PBMCs of male T2DM. LVDD = Left ventricular diastolic dysfunction. PBMCs = Peripheral blood mononuclear cells. Data are presented as means ± SD, data were tested by using Analysis of Covariance, age and HbA1C were adjusted, and followed by LSD post hoc analysis. *P* value <.05 was considered statistically significant. (Figure A. DM control n = 30, DM + LVDD1 n = 22, DM + LVDD2 n = 22; Figures B and C. DM control n = 15, DM + LVDD1 n = 11, DM + LVDD2 n = 11)

The GRK2 mRNA level in PBMCs of diabetes patients with LVDD was significantly increased than that of the diabetic controls without LVDD with age and HbA1C adjusted (Figure 6A). There was no significant difference in GRK2 mRNA level in PBMCs between DM + LVDD1 group and DM + LVDD2 group and so as GRK2‐mRNA level in female T2DM and male T2DM (Figure 6B,C).

## 4 DISCUSSION

HF in diabetic patients is associated with not only coronary artery diseases but also DCM, which is described as a cardiometabolic disease. Diastolic dysfunction together with concentric cardiac hypertrophy is considered the first hallmark of DCM. Although the mechanism of DCM is not fully understood, diastolic dysfunction and remodeling of ventricular concentric hypertrophy might be associated with metabolic damage in diabetes.^20^, ^21^

The disappointment of HF outcome in Cardiovascular Outcome trials^22^, ^23^, ^24^ in patients with T2DM to date suggests that the mild/moderate DCM patients selected for these studies are very difficult to recover completely. Therefore, DCM therapeutics may have significant disease‐modifying properties only if administered during the preclinical or prodromal stages of the disease. From this view, the reliable diagnosis methods to identify people in these incipient stages of the disease will be fundamental. Herein, the goal of the present study was to explore a new potential biomarker‐GRK2, for early diagnosis of DCM.

In the present study, the diagnosis of the early stage of DCM in 12‐week‐old diabetic mice was based on pathophysiology and ultrasonographic left ventricular hypertrophy, which was consistent with previous studies reporting that early stage of DCM characterized by left ventricular concentric hypertrophy pathophysiology. It will be more profound if the diastolic function was presented. However, the transmitral E and A waves fuse at heart rates >500 beats/min, E/A ratio could only be assessed in mice with heart rates ≤500 beats/min.^25^, ^26^ And the heart rate of 12‐week‐old mice was too high to detect the E/A ratio. The results showed a significant increase in GRK2 expression in myocardial tissue of diabetic mice at 12 weeks of age along with increases of cardiomyocyte apoptosis and myocardial fibrosis and ventricular wall thickening, which can be considered as the early stage of DCM.^27^, ^28^ GRK3 and GRK5 were found in the left ventricular myocardium of mice, but there was no significant difference compared to nondiabetic controls at the corresponding age. Because previous studies revealed that myocardial GRK2 levels are mirrored by the GRK2 levels in PBMCs of HF patients, we detected the GRK2 expression in PBMCs of T2DM patients with LVDD. LVDD also can be considered as the manifestations of the early stage of diabetic cardiomyopathy because other heart diseases such as coronary heart diseases, dilated cardiomyopathy, and hypertension were excluded in this study. The results revealed that the expression of GRK2 in PBMCs was elevated in T2DM patients with LVDD compared to T2DM patients without LVDD. Taken together, the results mentioned previously indicated that GRK2 expression increased not only in the cardiomyocytes of DCM but also in PBMCs of patients with LVDD, thus GRK2 might serve as a biomarker of early DCM.

Although the possible underlying mechanisms are not fully understood, GRK2 might serve as a promising biomarker of early DCM. First, our present study revealed that GRK2 expression increased in both mice myocardium tissue and patient PBMCs with early diabetic cardiomyopathy. Second, studies show that GRK2 expression is not only related to energy metabolism disorder of diabetes but also plays a key role in diastolic function and systolic function. Previous studies reveal that GRK2 has a key role in the systolic and diastolic function of the heart by regulating β‐adrenergic receptor‐mediated signaling and other signals. Studies also showed that GRK2 upregulation was associated with the dysfunction of β‐adrenergic receptor‐mediated signaling and decreasing of heart contractility in HF.^29^ Conversely, cardiac‐specific GRK2 knock‐out in mice caused a decrease in circulating catecholamine, improvement in cardiac function, and reservation in β‐adrenergic signaling.^30^ Thus, GRK2 expression in the heart appears to be related to cardiac contractile function via β‐adrenergic signaling.^20^, ^21^ Based on the previous studies and the results of the present study, this leads to the thought that GRK2 upregulation in the heart might be associated with diastolic dysfunction and possibly via regulating β‐adrenergic receptor‐mediated signaling in diabetes,^31^ although further studies needed to confirm that. Additionally, upregulated GRK2 not only related to β‐adrenergic receptor‐mediated signaling dysfunction but also diminished contractile responsiveness of the heart to positive inotropes, as it abrogates the pro‐contractile signaling of angiotensin II type 1 receptors, etc.^32^

Other evidence of GRK2 related to DCM was provided by inhibiting the GRK2 expression. Inhibition of GRK2 increased oxygen consumption rates and ATP production,^33^ reduced the degree of myocardial remodeling,^34^ and abolished the AVP‐induced IL‐6 production and NF‐κB activation.^35^ Moreover, a very recent paper showed that GRK2 inhibition can be safely achieved with beneficial effects in diabetes, and beyond its effects on the glycemic profile, exerted the anti‐inflammatory and antioxidative effect on the heart, which indicated a potential therapeutic action on DCM.^36^

In addition to β‐adrenergic receptor‐mediated signaling, other mechanisms including insulin resistance, fatty acid oxidation, and cardiomyocyte oxidative stress might be involved in the relation between GRK2 and DCM. What is more, some studies revealed that the sympathetic nervous system hyperactivity or the autonomic imbalance is some of the potential mechanisms involved in the development of diabetic cardiomyopathy, because significant reductions in coronary blood flow were observed in diabetic patients with cardiac autonomic neuropathy.^37^

The present study complemented a growing list of candidate biomarkers for DCM, such as NT‐proBNP, ANP, Cardiotrophin‐1, IGFBP‐7, and GRK2. Different from the elevated GRK2 expression, elevated NT‐proBNP^38^ and ANP^39^ levels suggest the advanced stage of cardiomyopathy, especially for heart failure; plasma cardiotrophin‐1 expression elevated in diabetes patients with cardiac hypertrophy and systolic dysfunction^40^; and IGFBP‐7 expression was revealed to be related to insulin resistant, cardiac hypertrophy, and cardiac fibrosis.^41^ These biomarkers represent several putative mechanistic pathways underlying DCM progression. However, expression of GRK2 and other biomarkers mentioned previously are not exclusive for the DCM, and high levels of biomarkers have also been found in other different cardiomyopathies and stress.

A caveat to our findings is that the sample sizes were limited. Future efforts will involve improving the test of the GRK2 level in PBMCs, and a longitudinal study with adequate power for gauging the utility of GRK2 for the onset of DCM. We should also keep in mind that the animal study only included male mice but no female mice included; what is more, the age of mice is much younger than that of the patient population; that is because db/db mice developed diabetes at a much younger age than the patients of this study. Although the mice are obesity whereas the patients in this study have normal body mass index scores, our clinical data and a previous study have revealed that body weight is not related to diabetic cardiomyopathy significantly. And we should also keep in mind that the diastolic function and GRK2 expression in PMBCs of 12‐week‐old mice were lacking in the present study. These data only provided important clues and possibility that GRK2 might function as a biomarker for the diagnosis of DCM early stage; however, further research is still needed.

## 5 CONCLUSIONS

GRK2 expression increased in the myocardial tissue and the PBMCs at the early stage of DCM. These data support further research on the role of GRK2 as the clinical biomarker of early DCM.

## CONFLICT OF INTEREST

The authors declare that they have no competing interests.

## AUTHORS' CONTRIBUTIONS

Hongmei Chen conducted a literature search, assisted with study design and data interpretation, draft the manuscript. Shuiqing Lai, Xiaoying Fu, Shufen Yang, and Shuting Zhang participated in study design, conducted the experiment, data collection, data analysis, and draft the manuscript. Mengzhen Zhang and Qiuxiong Lin conducted the experiment, participated in data acquisition, and contributed to data analysis and interpretation. All authors read and approved the final manuscript.

## References

[1] Thrainsdottir IS , Aspelund T , Thorgeirsson G , et al. The association between glucose abnormalities and heart failure in the population‐based Reykjavik study. Diabetes Care. 2005;28:612‐616. 15735197 10.2337/diacare.28.3.612

[2] Cheng YJ , Imperatore G , Geiss LS , et al. Trends and disparities in cardiovascular mortality among U.S. adults with and without self‐reported diabetes, 1988‐2015. Diabetes Care. 2018;41:2306‐2315. 30131397 10.2337/dc18-0831 PMC7849201

[3] van Melle JP , Bot M , de Jonge P , de Boer RA , van Veldhuisen DJ , Whooley MA . Diabetes, glycemic control, and new‐onset heart failure in patients with stable coronary artery disease: data from the heart and soul study. Diabetes Care. 2010;33:2084‐2089. 20805280 10.2337/dc10-0286 PMC2928369

[4] MacDonald MR , Petrie MC , Varyani F . et al; CHARM InvestigatorsImpact of diabetes on outcomes in patients with low and preserved ejection fraction heart failure: an analysis of the candesartan in heart failure: assessment of reduction in mortality and morbidity (CHARM) programme. Eur Heart J. 2008;29:1377‐1385. 18413309 10.1093/eurheartj/ehn153

[5] Venskutonyte L , Jarnert C , Rydén L , Kjellström B . Longitudinal development of left ventricular diastolic function in patients with type 2 diabetes. Diabetes Care. 2014;37:3092‐3097. 25193530 10.2337/dc14-0779

[6] Von Bibra H , Thrainsdottir IS , Hansen A , Dounis V , Malmberg K , Rydén L . Tissue Doppler imaging for the detection and quantitation of myocardial dysfunction in patients with type 2 diabetes mellitus. Diab Vasc Dis Res. 2005;2:24‐30. 16305069 10.3132/dvdr.2005.002

[7] Iaccarino G , Barbato E , Cipolletta E , et al. Elevated myocardial and lymphocyte GRK2 expression and activity in human heart failure. Eur Heart J. 2005;26:1752‐1758. 16055494 10.1093/eurheartj/ehi429

[8] Hata JA , Williams ML , Schroder JN , et al. Lymphocyte levels of GRK2 (betaARK1) mirror changes in the LVAD‐supported failing human heart: lower GRK2 associated with improved beta‐adrenergic signaling after mechanical unloading. J Card Fail. 2006;12:360‐368. 16762799 10.1016/j.cardfail.2006.02.011

[9] Bonita RE , Raake PW , Otis NJ , et al. Dynamic changes in lymphocyte GRK2 levels in cardiac transplant patients: a biomarker for left ventricular function. Clin Transl Sci. 2010;3:14‐18. 20443948 10.1111/j.1752-8062.2010.00176.x PMC3018749

[10] Rengo G , Pagano G , Filardi PP , et al. Prognostic value of Lymphocyte G protein‐coupled receptor Kinase‐2 protein levels in patients with heart failure. Circ Res. 2016;118:1116‐1124. 26884616 10.1161/CIRCRESAHA.115.308207 PMC4818176

[11] Liggett SB , Lymphocyte GRK . Levels as biomarkers in heart failure. Eur Heart J. 2005;26:1695‐1696. 16055498 10.1093/eurheartj/ehi355

[12] Santulli G , Campanile A , Spinelli L , et al. G protein‐coupled receptor kinase 2 in patients with acute myocardial infarction. Am J Cardiol. 2011;107:1125‐1130. 21296320 10.1016/j.amjcard.2010.12.006

[13] Rengo G , Pagano G , Paolillo S , et al. Impact of diabetes mellitus on lymphocyte GRK2 protein levels in patients with heart failure. Eur J Clin Invest. 2015;45:187‐195. 25545706 10.1111/eci.12395

[14] Yu XY , Chen HM , Liang JL , et al. Hyperglycemic myocardial damage is mediated by proinflammatory cytokine: macrophage migration inhibitory factor. PLoS One. 2011;6:e16239. 10.1371/journal.pone.0016239 PMC3026813 21283592

[15] Chen HM , Lin QX , Tan HH , Yang HZ , Yu XY . High glucose up‐regulates GRK2 gene expression in H9C2 cardiomyoblasts in vitro. Nan Fang Yi Ke Da Xue Xue Bao. 2010;30:472‐474. 20335112

[16] Bowden MA , Tesch GH , Julius TL , Rosli S , Love JE , Ritchie RH . Earlier onset of diabesity‐induced adverse cardiac remodeling in female compared to male mice. Obesity (Silver Spring). 2015;23:1166‐1177. 25959739 10.1002/oby.21072

[17] Alex L , Russo I , Holoborodko V , Frangogiannis NG . Characterization of a mouse model of obesity‐related fibrotic cardiomyopathy that recapitulates features of human heart failure with preserved ejection fraction. Am J Physiol Heart Circ Physiol. 2018;315:H934‐934H949. 30004258 10.1152/ajpheart.00238.2018 PMC6230908

[18] Hanif W , Alex L , Su Y , et al. Left atrial remodeling, hypertrophy, and fibrosis in mouse models of heart failure. Cardiovasc Pathol. 2017;30:27‐37. 28759817 10.1016/j.carpath.2017.06.003 PMC5592139

[19] Yu XY , Lin SG , Wang XM , et al. Evidence for coexistence of three beta‐adrenoceptor subtypes in human peripheral lymphocytes. Clin Pharmacol Ther. 2007;81:654‐658. 17361123 10.1038/sj.clpt.6100154

[20] Rijzewijk LJ , Jonker JT , van der Meer RW , et al. Effects of hepatic triglyceride content on myocardial metabolism in type 2 diabetes. J Am Coll Cardiol. 2010;56:225‐233. 20620743 10.1016/j.jacc.2010.02.049

[21] Ng AC , Delgado V , Bertini M , et al. Myocardial steatosis and biventricular strain and strain rate imaging in patients with type 2 diabetes mellitus. Circulation. 2010;122:2538‐2544. 21126971 10.1161/CIRCULATIONAHA.110.955542

[22] Cavender MA , Steg PG , Smith SC Jr . et al; REACH Registry InvestigatorsImpact of diabetes mellitus on hospitalization for heart failure, cardiovascular events, and death: outcomes at 4 years from the reduction of Atherothrombosis for continued health (REACH) registry. Circulation. 2015;132:923‐931. 26152709 10.1161/CIRCULATIONAHA.114.014796

[23] MacDonald MR , Petrie MC , Hawkins NM , et al. Diabetes, left ventricular systolic dysfunction, and chronic heart failure. Eur Heart J. 2008;29:1224‐1240. 18424786 10.1093/eurheartj/ehn156

[24] Pocock SJ , Wang D , Pfeffer MA , et al. Predictors of mortality and morbidity in patients with chronic heart failure. Eur Heart J. 2006;27:65‐75. 16219658 10.1093/eurheartj/ehi555

[25] Semeniuk LM , Severson DL , Kryski AJ , Swirp SL , Molkentin JD , Duff HJ . Time‐dependent systolic and diastolic function in mice overexpressing calcineurin. Am J Physiol Heart Circ Physiol. 2003;284:H425‐H430. 12388248 10.1152/ajpheart.00546.2002

[26] Taffet GE , Hartley CJ , Wen X , Pham T , Michael LH , Entman ML . Noninvasive indexes of cardiac systolic and diastolic function in hyperthyroid and senescent mouse. Am J Physiol. 1996;270:H2204‐H2209. 8764275 10.1152/ajpheart.1996.270.6.H2204

[27] Yue P , Arai T , Terashima M , et al. Magnetic resonance imaging of progressive cardiomyopathic changes in the db/db mouse. Am J Physiol Heart Circ Physiol. 2007;292:H2106‐H2118. 17122193 10.1152/ajpheart.00856.2006

[28] Bugger H , Abel ED . Rodent models of diabetic cardiomyopathy. Dis Model Mech. 2009;2:454‐466. 19726805 10.1242/dmm.001941

[29] Theilade J , Strøm C , Christiansen T , Haunsø S , Sheikh SP . Differential G protein receptor kinase 2 expression in compensated hypertrophy and heart failure after myocardial infarction in the rat. Basic Res Cardiol. 2003;98:97‐103. 12607131 10.1007/s00395-003-0395-x

[30] Lymperopoulos A , Rengo G , Gao E , Ebert SN , Dorn GW 2nd , Koch WJ . Reduction of sympathetic activity via adrenal‐targeted GRK2 gene deletion attenuates heart failure progression and improves cardiac function after myocardial infarction. J Biol Chem. 2010;285:16378‐16386. 20351116 10.1074/jbc.M109.077859 PMC2871505

[31] Wang Q , Liu Y , Fu Q , et al. Inhibiting insulin‐mediated β2‐adrenergic receptor activation prevents diabetes‐associated cardiac dysfunction. Circulation. 2017;135:73‐88. 27815373 10.1161/CIRCULATIONAHA.116.022281 PMC5302024

[32] Lymperopoulos A , Rengo G , Koch WJ . GRK2 inhibition in heart failure: something old, something new. Curr Pharm des. 2012;18:186‐191. 22229578 10.2174/138161212799040510

[33] Sato PY , Chuprun JK , Grisanti LA , et al. Restricting mitochondrial GRK2 post‐ischemia confers cardioprotection by reducing myocyte death and maintaining glucose oxidation. Sci Signal. 2018;11(560):eaau0144. 10.1126/scisignal.aau0144 PMC6463290 30538174

[34] Woodall MC , Woodall BP , Gao E , Yuan A , Koch WJ . Cardiac fibroblast GRK2 deletion enhances contractility and remodeling following ischemia/reperfusion injury. Circ Res. 2016;119:1116‐1127. 27601479 10.1161/CIRCRESAHA.116.309538 PMC5085864

[35] Xu F , Sun S , Wang X , Ni E , Zhao L , Zhu W . GRK2 mediates arginine vasopressin‐induced Interleukin‐6 production via nuclear factor‐κB signaling neonatal rat cardiac fibroblast. Mol Pharmacol. 2017;92:278‐284. 28193640 10.1124/mol.116.107698

[36] Cipolletta E , Gambardella J , Fiordelisi A , et al. Antidiabetic and Cardioprotective effects of pharmacological inhibition of GRK2 in db/db mice. Int J Mol Sci. 2019;20:1492. 10.3390/ijms20061492 PMC6470575 30934608

[37] Kasznicki J , Drzewoski J . Heart failure in the diabetic population ‐ pathophysiology, diagnosis and management. Arch Med Sci. 2014;10:546‐556. 25097587 10.5114/aoms.2014.43748 PMC4107260

[38] Korkmaz‐Icöz S , Lehner A , Li S , et al. Left ventricular pressure‐volume measurements and myocardial gene expression profile in type 2 diabetic Goto‐Kakizaki rats. Am J Physiol Heart Circ Physiol. 2016;311:H958‐958H971. 27521423 10.1152/ajpheart.00956.2015

[39] Inoue Y , Kawai M , Minai K , et al. The impact of an inverse correlation between plasma B‐type natriuretic peptide levels and insulin resistance on the diabetic condition in patients with heart failure. Metabolism. 2016;65:38‐47. 26892514 10.1016/j.metabol.2015.09.019

[40] Gamella‐Pozuelo L , Fuentes‐Calvo I , Gómez‐Marcos MA , et al. Plasma cardiotrophin‐1 as a marker of hypertension and diabetes‐induced target organ damage and cardiovascular risk. Medicine (Baltimore). 2015;94:e1218. 10.1097/MD.0000000000001218 PMC4554114 26222851

[41] Gandhi PU , Gaggin HK , Sheftel AD , et al. Prognostic usefulness of insulin‐like growth factor‐binding protein 7 in heart failure with reduced ejection fraction: a novel biomarker of myocardial diastolic function. Am J Cardiol. 2014;114:1543‐1549. 25248814 10.1016/j.amjcard.2014.08.018

